# Experimental Evidence of the Long-Term Survival of Infective African Swine Fever Virus Strain Ba71V in Soil under Different Conditions

**DOI:** 10.3390/pathogens11060648

**Published:** 2022-06-04

**Authors:** Jana Prodelalova, Lenka Kavanova, Jiri Salat, Romana Moutelikova, Sarka Kobzova, Magdalena Krasna, Petra Vasickova, Bronislav Simek, Petr Vaclavek

**Affiliations:** 1 Veterinary Research Institute, Hudcova 70, 602 00 Brno, Czech Republic; lenka.kavanova@vri.cz (L.K.); jiri.salat@vri.cz (J.S.); romana.moutelikova@vri.cz (R.M.); sarka.kobzova@vri.cz (S.K.); magdalena.krasna@vri.cz (M.K.); petra.vasickova@vri.cz (P.V.); 2 State Veterinary Institute Jihlava, Rantirovska 20, 586 01 Jihlava, Czech Republic; simek@svujihlava.cz (B.S.); vaclavek@svujihlava.cz (P.V.)

**Keywords:** ASFV, field isolates, stability, hemadsorption, infectivity testing

## Abstract

The survival of African swine fever virus (ASFV) on different matrices and its infectivity in wild as well as domestic swine is still a matter of interest. ASFV is resistant to environmental effects; this fact is enhanced by the presence of organic material. Therefore, the aim of this work was to determine the ability of laboratory ASFV to survive in soil at different temperatures (4 and 22 °C) and with and without the presence of blood using culture procedures. The suitability of the procedure for determining the viability and titre of the ASFV field strain by the hemadsorption method was also verified, when a higher decrease in virus infectivity in the case of clay compared with peat was demonstrated. The stability of the virus was clearly temperature-dependent, the infectious virus was detected after 112 days, and the viral DNA was still detected in the matrix 210 days after inoculation in a relatively high and stable concentration (between 10^6^ and 10^7^ genome equivalents/mL). Based on this knowledge, soil and other environmental samples could provide rapid and reliable information on the disease outbreak and serve as indicators of the risk posed by the affected locality.

## 1. Introduction

Recently, the African swine fever virus (ASFV), an aetiological agent of African swine fever, has become a severe threat in pig industry worldwide. ASFV affects members of the family *Suidae*, including both domestic pigs and wild boars (*Sus scrofa*) [1]. The epidemiology of ASFV consists of four transmission cycles. Three epidemiological cycles have been observed in sub-Saharan Africa; namely, (1) the sylvatic cycle, which comprises soft ticks of the genus *Ornithodoros* and common warthog (*Phacochoerus africanus*); (2) the pig–tick cycle, and (3) the anthropogenic cycle [2,3]. Since 2014, when ASFV reached the European Union, a previously undescribed wild boar–habitat epidemiologic cycle has been noticed based on reported outbreak data. The cycle includes both direct and indirect transmission. Whereas direct transmission obviously depends on contact between sick and healthy individuals, indirect transmission is based on environments contaminated by ASFV-positive wild boar carcasses [2]. Recently, the role of both domestic pig and wild boar carcasses has been analysed. In general, they were recognized as a source of ASFV particles capable of causing a disease in wild boars [4,5]. Probst et al. have described wild boars directly contacting their dead conspecifics in different stages of decomposition, mainly by rooting near or underneath the carcases, sniffing, poking, and chewing bare bones [6]. However, no signs of cannibalism have been observed. Similar observations were recorded during the study of Masiulis et al. [7]. Moreover, no preference between domestic pig and wild boar carcasses is described in this work. On the contrary, during the study of Cukor et al., cannibalism between the wild boars and wild boar carcasses in the winter season was clearly noticed [8]. Nevertheless, the risk of ASFV transmission is not only in cannibalism but also in manipulating soil contaminated by liquids or blood residues from decomposing carcasses [9]. The ASFV is considered to be extremely stable in the environment; it is highly resistant especially to temperature and acid pH. Particles were stable in serum-free medium at pH 4–10. Another highly important characteristic in terms of virus survival is the stability of the virus against proteases and nucleases [1]. The cause of the high resistance of viral particles lies in the extremely complex structure of the virion [10]. ASFV is the sole member of the *Asfarviridae* family and belongs to the group of large nucleocytoplasmic DNA viruses. The outstanding feature of the infectious ASF virion is its multilayer architecture. An ASFV genome-containing nucleoid is surrounded by two distinct icosahedral protein capsids and two lipoprotein membranes [11]. Moreover, the presence of organic matter such as blood, serum, and uncooked meat or meat products increases the stability of the virus [1].

In this work, we aimed to obtain a more complete picture of the ability of ASFV to survive in soil under different conditions. In the first part of the experiment, a laboratory ASFV strain Ba71V that is able to grow and produce visible cytopathic effects on a VERO cell line was used. Furthermore, we developed a method suitable for the detection of infective ASFV particles in the soil. To verify the procedure, we used recent field isolate, which does not produce cytopathic effects (CPE) on established cell lines. Thus, we had to use primary porcine alveolar macrophages and quantify the virus by hemadsorption test.

## 2. Materials and Methods

### 2.1. ASFV Ba71V Stock Preparation

ASFV strain Ba71V stocks obtained from the European Union Reference Laboratory for African swine fever (EURL-ASF) were used in the study. The Ba71V strain was propagated on VERO cells. Dulbecco’s Modified Eagle Medium High Glucose (DMEM; Biosera, Nuaille, France) supplemented with 10% (*v*/*v*) gamma-irradiated foetal bovine serum (FBS; HyClone Laboratories, Cramlington, UK) was used for VERO cultivation. A confluent monolayer of cells (cultivated for a maximum of 24 h) was inoculated with virus suspension. After 1 h of virus adsorption, DMEM with FBS (2% *v*/*v*) was added, and infected cells were cultivated at 37 °C under 5% CO_2_. An extensive cytopathic effect (CPE) was observed in the cell culture at six days post infection (DPI). Next, the cells were frozen at −80 °C and thawed twice. Cell debris was removed by centrifugation (1500× *g*, 15 min, 4 °C). Then, viral particles were concentrated employing Pierce™ Protein Concentrator (30K MWCO, 20–100 mL; Thermo Scientific, Rockford, IL, USA) following the manufacturer’s instructions to obtain virus stock for survival testing. The prepared virus suspension was stored at −80 °C until further use. The virus concentration was determined by titration assay on VERO cells, and the final concentration was 7.5 × 10^7^ TCID_50_/mL.

### 2.2. ASFV Field Isolates Stocks Preparation

Field isolates were subcultured successfully on primary porcine alveolar macrophages (PAMs). PAMs were prepared according to Kavanová et al. [12]. Field strains were isolated from the PCR-positive spleens of two wild boars (sample Nos. 175576/17 and 103483/18) detected at the State Veterinary Institute Jihlava, Czech Republic, during ASFV outbreaks in 2017 and 2018. Briefly, 100 mg of spleen was homogenized using Tissue Stainer in sterile Dulbecco’s phosphate-buffered saline (DPBS; Biosera, Nuaille, France) to obtain a 10% suspension; next, ceramic beads (0.6–0.8 mm; Macherey-Nagel, Düren, Germany) were used to prepare suspension. Spleen homogenate was centrifuged (5000× *g*, 10 min), supernatant was filtered through 0.45 µm filter (TPP, Trasadingen, Switzerland), diluted 50× in DMEM supplemented with 10% (*v*/*v*) FBS and 1% (*v*/*v*) Antibiotic-Antimycotic 100× (Biosera, Nuaille, France). Four wells (24-well tissue culture test plate, TPP, Trasadingen, Switzerland) of PAMs (5 × 10^5^ cells per well) were infected with 200 µL of prepared inoculum. After 1 h of absorption, 1 mL of DMEM (10% FBS, 1% ATB) was added, and infected cells were cultivated at 37 °C under 5 % CO_2_. Infected cells were observed daily for any microscopical changes. After 5 days, CPE was observed in comparison with non-infected control cells, and the culture plates were freeze-thawed for further passaging. The second passage was performed under the same conditions as the first passage. The following passage was performed on cell culture flasks (TPP, Trasadingen, Switzerland) (5 × 10^6^ PAMs per flask with growth surface of 25 cm^2^). The cells were inoculated with 500 µL of virus harvest (4× diluted freeze-thawed second passage), and after 20 min of absorption, 10 mL of DMEM (10% FBS, 1% ATB) was added. Infected cells were incubated at 37 °C in 5% CO_2_. CPE was observed after 6–7 days of cultivation. Thereafter, the cells were freeze-thawed, and the aliquots with virus harvest were frozen in −80 °C until use.

### 2.3. Whole-Genome Sequencing of ASFV Field Isolates

To work with well-defined virus, the stock virus of the Czech ASFV isolates (samples No. 175576/17 and 103483/18) prepared for further experiments was characterized by next-generation sequencing. DNA from the virus aliquots was isolated with the use of a QIAamp DNA Mini Kit (Qiagen, Hilden, Germany). The DNA library preparation, Illumina sequencing, and nucleotide sequence mapping to ASFV reference sequences were performed by a commercial provider (SeqMe, Dobříš, Czech Republic). The numbers of 250 bp pair-end reads per sample were 1,386,592 and 1,309,234 reads, and the percentages of mapped reads 9.5% and 11% (for 175576/17 and 103483/18, respectively). Nucleotide sequences for each ASFV isolate were assembled into contigs using the high-performance graphical viewer Tablet [13].

The sequences of genes coding main structural proteins p30, p54, and p70 for both ASFV field isolates were deposited in the GenBank. They received accession numbers MW889885–MW889890.

### 2.4. Long-Term Survival Assay with Ba71V Strain

Ba71V strain is able to produce CPE on a commonly used VERO cell line, so it is easy to quantify it using titration. Thus, we used this method for testing the long-term survival of ASFV in soil under different conditions. To ensure defined identical conditions during all sampling times, sand-clay soil was sterilized by autoclaving at 121 °C for 15 min and divided into 1 g aliquots in 15-mL centrifuge tubes (TPP, Trasadingen, Switzerland). The pH of soil samples was determined in water extracts using a soil water ratio of 1:2 with a glass electrode and a direct-reading pH meter. All samples of sterile soil were inoculated with 500 µL of Ba71V virus strain (7.5 × 10^7^/mL): (1) in DMEM with FBS (2% *v*/*v*), which represents less biological soiling, and (2) in DMEM with FBS (2% *v*/*v*) and 10% defined commercial sheep erythrocytes (Labmedia Servis, Jaroměř, Czech Republic) to simulate more biological soiling. Thereafter, inoculated samples were stored in two different conditions until use: (1) at room temperature protected from light (22 °C) and (2) at 4 °C. All samples were tested in duplicate.

### 2.5. Detection of Infectious ASFV Ba71V Particles in Soil

The virus (Ba71V strain) was isolated from the inoculated soil samples (described in paragraph 2.4) at 0, 1, 14, 28, 42, 56, 63, 70, 112, 126, 147, 176, and 210 days post inoculation (DPI). The soil inoculated with virus was removed from storage, 10 mL of DMEM (10% FBS, 1% ATB) was added, and samples were shaken vigorously for 20 min. The samples were centrifuged at 5000× *g* for 15 min, and the supernatants were filtered with 0.45 µm syringe filter (TPP, Trasadingen, Switzerland) and placed in a Pierce™ Protein Concentrator (30K MWCO, 5–20 mL; Thermo Scientific, Rockford, IL, USA). The supernatants were concentrated to original inoculum volume (500 µL) by centrifugation at 5000× *g* for approximately 15 min. The virus concentration was determined by titration assay on the VERO cells. The limit of detection in this assay is 10^1.5^ TCID_50_/mL.

### 2.6. Detection of ASFV Ba71V Genome in Soil

Viral DNA was extracted from 100 μL of every concentrated supernatant originating from soil (see paragraph 2.5) with the use of the QIAamp Viral RNA Mini Kit (Qiagen, Hilden, Germany) and eluted into 60 μL of PCR-grade water, according to the manufacturer’s instructions. 

Based on the standard operating procedure of the European Reference Laboratory for African swine fever, real-time PCR (qPCR) was introduced and optimized to detect and quantify the ASFV genome [14]. An internal amplification control (IAC) was incorporated into the assay [15]. The reaction mix contained 10 µL of LightCycler 480 Probes Master (Roche Molecular Diagnostics, Manheim, Germany); 10 pmol each of King-s, King-a, IAC-F, and IAC-R primers; 4 pmol of King-probe; 2 pmol of IAC-probe, 10^3^ copies of IAC DNA, and 5 µL template DNA. To avoid possible carry-over contamination, 0.2 U of Uracil DNA Glycosylase (Roche) was used in each qPCR reaction. The optimised duplex qPCR was run in a total volume of 20 µL. Amplification and fluorescence detection were performed on the LightCycler 480 (Roche) using 96-well PCR plates under the following conditions: initial denaturation at 95 °C for 4 min and 45 cycles of 95 °C for 10 s, then 58 °C for 30 s. Consequently, the DNA standards (plasmid; concentration 1 × 10^6^, 1 × 10^5^, 1 × 10^4^, 1 × 10^3^, 1 × 10^2^ and 1 × 10^1^ copies/µL) were used to construct a gradient, which served for the quantitation of ASFV (genomic equivalents; GE) and as positive controls. The analysis of results was carried out using the “Fit Points Method” option of the LightCycler 480 Software release 1.5.0 (version 1.5.0.39, Creator, Roche Molecular Systems, Inc., Basel, Switzerland).

### 2.7. Recovery and Quantification of Infectious Field Isolate Virions from Different Soil Types 

In the next step, we decided to demonstrate the efficiency of isolation of infectious virus particles from different soil types using ASFV field isolate instead of laboratory Ba71V strain. In contrast to Ba71V, field ASFV isolate does not produce the cytopathic effect (CPE) on established cell lines, and conversely, Ba71V strain was not able to produce typical signs of hemadsorption, e.g., rosettes. Therefore, we had to use primary porcine cells (alveolar macrophages) and field isolate 175576/17 and quantify the virus by hemadsorption test.

The infectivity of ASFV isolate was tested after incubation in two different matrices: (1) soil (pH 7.78) or (2) peat (pH 3.85). The amount of 500 µL of virus (1.5 × 10^7^ HAD_50_/mL) was added into 1 g of matrix and incubated for 15 min at room temperature. The titre of the field virus isolate was assessed by hemadsorption test based on the EURL-ASF standard operating procedure [16]; the protocol was slightly modified using 384-well plates (SPL Life Sciences, Pocheon-City, Korea), which enabled significantly reducing the amounts of PAMs and porcine erythrocytes used in the assay. Consequently, 50% hemadsorption doses (HAD_50_/mL) were determined. Alveolar macrophages were seeded on a 384-well plate at a concentration of 3×10^4^ per well in DMEM (10% FBS, 1% ATB) and incubated overnight at 37 °C in 5% CO_2_. ASFV was diluted (10fold serial dilution) using macrophage medium (DMEM; 10% FBS, 1% ATB), and 25 µL of each dilution was transferred to five wells of the micro-titre plate. The amount of 25 µL of 0.1% porcine erythrocytes (isolated from porcine heparinized blood by Histopaque-1077 (Sigma-Aldrich) gradient) was added after 20 min of adsorption. Erythrocytes were diluted in DMEM (10% FBS, 1% ATB). The presence of the virus was assessed by hemadsorption 7 days after inoculation, and virus titres were calculated by the Reed and Muench method [17]. Non-infected PAMs with 0.1% porcine erythrocytes were used as a control of unspecific hemadsorption reactions.

## 3. Results

### 3.1. Genetic Characterization of ASFV Field Isolates

Both field isolates, 175576/17 and 103473/18, were characterized by NGS sequencing and the analysis of the gene coding of three major ASFV antigens, the polypeptides p30, p54, and p72, after three passages on PAM cells. The NGS analysis showed no differences between the two field ASFV isolates, and all three main polypeptide sequences of both field isolates were identical to the corresponding genome regions of the Czech strain 2017/1 as well as the strain Georgia 2008/2 (GenBank No. LR722600 and MH910496.1, respectively). Thus, further experiments with field ASFV were conducted only with 175576/17 isolate.

### 3.2. Long-Term Survival of ASFV Ba71V Strain in Soil under Different Temperatures

**Figure 1:**
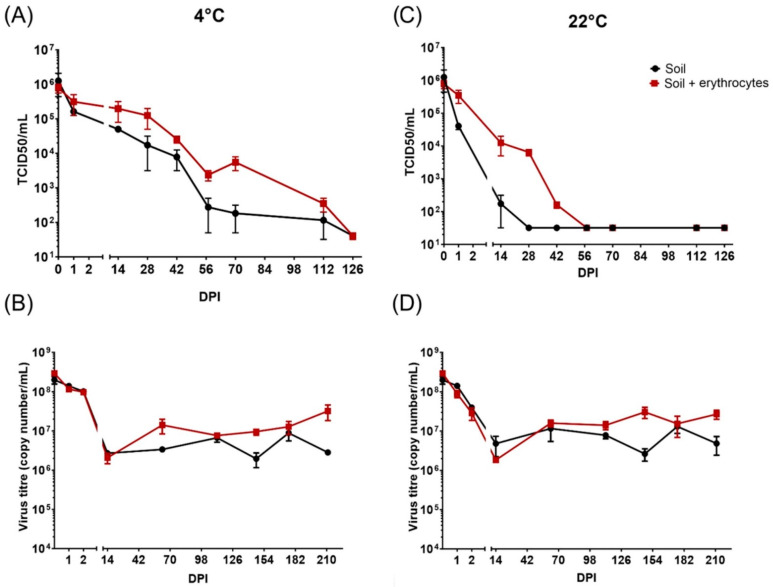
The detection of the presence of infectious Ba71V ASFV particles (**A**,**C**) and nucleic acid (**B**,**D**) in soil at 4 °C (**A**,**B**) and at 22 °C (**C**,**D**). Data are expressed as mean ± SE, and the experiment was performed in duplicate. The limit of detection of infectious particles (**A**,**C**) is 10^1.5^ TCID_50_/mL.

The virus (Ba71V) was isolated from soil stored under different conditions at regular intervals, and the virus concentration was determined by titration assay on VERO cells. The samples of soil were inoculated with 500 µL of virus (7.5 × 10^7^ TCID_50_/mL) per 1 g, and the isolation from the soil at day 0 (immediately after inoculation) showed a decrease in the virus load to 1.26 × 10^6^ TCID_50_/mL. The best ASFV stability was reported in the samples of soil incubated at 4 °C with an addition of sheep erythrocytes (Figure 1). Infectious ASFV (above the detection limit 10^1.5^ TCID_50_/mL) was still detected after 112 days of incubation in the samples with and without sheep erythrocytes at 4 °C (Figure 1A). However, the viable virus particles in the samples incubated at 22 °C without sheep erythrocytes were detected after 14 days of incubation. Longer viability of ASFV was observed in samples incubated at 22 °C with erythrocytes, where the virus was detectable 42 days after soil inoculation (Figure 1C). This part of the experiment was terminated at 126 days after inoculation when no infectious particles were detected in the samples of ASFV-inoculated soil.

The second part of the experiment was to determine the amount of ASFV nucleic acid by PCR. The ASFV genome was clearly detected under all tested conditions and time intervals. The main decrease was observed during the first 14 days after inoculation, from an initial concentration (day 0) of 1.40 × 10^8^ ASFV GE/mL to 2.70 × 10^6^ and 4.79 × 10^6^ ASFV GE/mL for incubation at 4 and 22 °C, respectively. At the following time points, the level of ASFV nucleic acid was stabilized and the amount varied between 10^6^ and 10^7^ GE/mL at both temperature conditions until the end of experiment (210 days after inoculation) (Figure 1B,D).

### 3.3. The Detection of Infectious Field Strain ASFV Particles from Soil and Peat Employing the Hemadsorption Test

**Figure 2:**
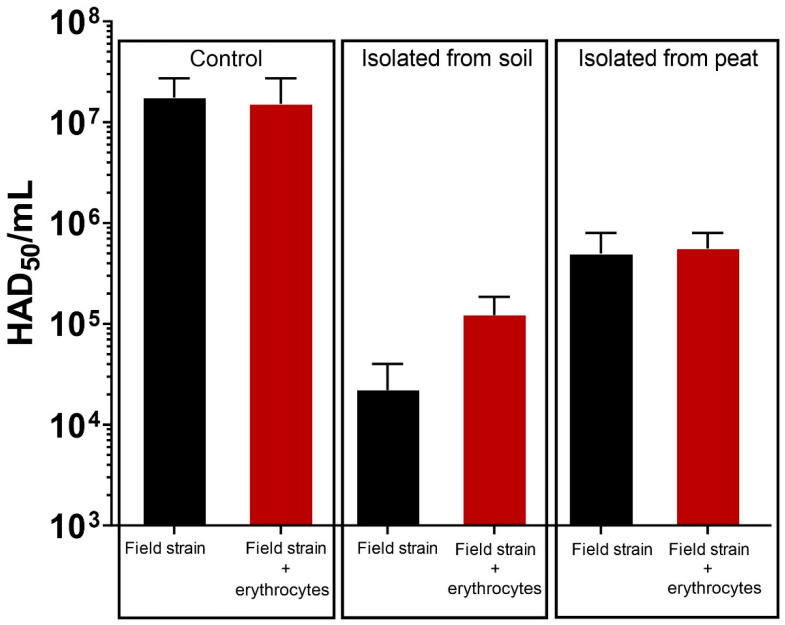
The isolation of the infectious field strain ASFV from different matrices (soil and peat) compared with its isolation from the control virus suspension. ASFV titres are expressed as mean ± SE of the HAD_50_ per ml, and the experiment was performed in duplicate.

The experiment verifies the possibility of field strain ASFV isolation from soil and peat. Mean titres of viable ASFV are presented in Figure 2. The virus remained infectious in both soil and peat. However, the titres decreased in samples isolated from soil (2 × 10^4^ and 1.2 × 10^5^ with and without erythrocytes, respectively) and peat (5 × 10^5^ and 5.5 × 10^5^ with and without erythrocytes, respectively) when compared with samples isolated directly from virus suspension (1.7 × 10^7^ and 1.6 × 10^7^ with and without erythrocytes, respectively).

## 4. Discussion

ASFV resistance and stability in the environment are the main prerequisites for its indirect transmission. It has been proved that ASFV is extremely resistant to environmental conditions and can survive many freeze-thaw cycles as well as a wide range of pH levels and temperatures [18]. Several studies were focused on the stability of ASFV in contaminated pork products, excretions or fomites [19,20,21]. Davies et al. described the detection of infectious ASFV in faeces for five days up to 12 °C. Similar data were obtained in experiments with urine. Increasing environmental temperature significantly shortened the survival time of ASFV [21]. In our study, the long-term survival of ASFV in soil was determined. Moreover, the assay enables the detection of ASFV in contaminated soil based on isolation of viral particles. Additionally, the ASFV cultivation method was optimized, which permits studying the role of contaminated soil in ASFV transmission. The novelty of our method of virus isolation from spiked soil is based on sample concentration by protein concentrators that could raise the sensitivity of the method. In addition, the method was used successfully for the isolation of infectious ASFV particles of Czech field isolate 175576/17 from both soil and peat and subsequent determination of virus titre by hemadsorption. 

We showed that the stability of the virus is clearly affected by the ambient temperature, similar to Davies et al. [21]. The virus was detectable at day 112 when stored at 4 °C, and the genome copy numbers were constant over 210 days post inoculation. The stability of ASFV at lower temperatures indicates the higher probability of spreading rapidly in wild boar populations during autumn-winter. Seasonality was observed in many European countries affected by ASF [22,23,24]. However, Carlson et al. reported that the level of infectivity could depend on the soil type, pH or organic material percentage [25]. Moreover, Mazur-Panasiuk and Woźniakowski discuss the importance of the sterility of the spiked matrix [26]. However, not only the abiotic factors affecting the infectivity of ASFV were studied. Hakobyan et al. investigated different invertebrate species as possible ASFV vectors [27]. Obviously, the methods of virus isolation differed from author to author, which could significantly affect the success rate of isolation. Therefore, our future study will be focused on determining the long-term viability of ASFV in peat whose pH could influence the stability of the virus. Similarly, the effect of sterilization of soil before inoculation will be assessed.

During the study, the substantial stability of ASFV genetic material has been proven. Zani et al. demonstrated relatively low virus contamination of soil surrounding wild boar carcasses buried in different levels of decomposition [28]. However, the number of analysed soil samples was limited, and the results showed the presence of the ASFV genome after 417 days of carcass burial. This finding corresponds with the results of our study: the main decrease of virus DNA (almost two orders of magnitude) was observed within 14 days of the experiment, and subsequently, stable quantities of virus DNA (between 10^6^ and 10^7^ GE/mL) were detected until 210 days post inoculation (end of the experiment) of soil samples. Unexpectedly, this stability of ASFV DNA was also observed at 22 °C. Lee et al. successfully detected the ASFV genome from mud and water samples from areas where ASFV-positive wild boar carcasses were found using the polyethylene glycol-based precipitation of viral particles [29]. Unfortunately, there is no possibility of verifying the method described in our study under field conditions due to the fact that the ASF outbreak was eradicated in the Czech Republic during 2018.

Based on this knowledge, soil and other environmental samples could provide rapid and reliable information on the disease outbreak and trends in animal population and can serve either as indicators of the risk posed by affected locality or as confirmations of area safety.

## Data Availability

Not applicable.
